# Black African and Caribbean British Communities’ Perceptions of Memory Problems: “We Don’t Do Dementia.”

**DOI:** 10.1371/journal.pone.0151878

**Published:** 2016-04-05

**Authors:** Sharne Berwald, Moïse Roche, Simon Adelman, Naaheed Mukadam, Gill Livingston

**Affiliations:** 1 Camden and Islington NHS Foundation Trust, London, United Kingdom; 2 Division of Psychiatry, UCL, London, United Kingdom; Taipei Veterans General Hospital, TAIWAN

## Abstract

**Objectives:**

We aimed to identify and explore the barriers to help-seeking for memory problems, specifically within UK Black African and Caribbean communities.

**Method:**

We purposively recruited participants from community groups and subsequent snowball sampling, to achieve a maximum variation sample and employed thematic analysis. Our qualitative semi-structured interviews used a vignette portraying a person with symptoms of dementia, and we asked what they or their family should do. We stopped recruiting when no new themes were arising.

**Results and significance:**

We recruited 50 people from a range of age groups, country of origin, time in the UK, religion and socio-economic background. Some of the barriers to presentation with dementia have been reported before, but others were specific to this group and newly identified. Many people recognised forgetfulness but neither that it could be indicative of dementia, nor the concept of dementia as applying to them. Dementia was viewed as a white person’s illness. Participants felt there was little point in consulting a doctor for forgetfulness. Many thought that seeing a GP was only for severe problems. Some said that their culture was secretive and highly valued privacy of personal affairs and therefore did not want to discuss what they regarded as a private and stigmatising problem with a GP. Participants did not appreciate their GP could refer to memory services who have more time and expertise. They were concerned about harm from medication and compulsory institutionalisation. Care should be from the family. Any intervention should emphasise the legitimacy of seeing a doctor early for memory concerns, that dementia is a physical illness which also occurs in the Black community, that help and time are available from memory services whose role is to prolong independence and support families in caring.

## Introduction

Black African and Caribbean elders (BACE) have a higher prevalence and earlier onset of dementia compared with the indigenous White UK population [1, 2]. Timely diagnosis of dementia is recommended throughout Europe [3, 4], as it maximises autonomy including the ability to make future choices while decisional capacity is present. It also enables people to make sense of what is happening and to receive appropriate services which may extend independence while living with dementia. Nonetheless, Black and Minority Ethnic (BME) groups throughout the developed world present later to dementia specialist services, frequently receiving a diagnosis when the disease is already well advanced [5, 6], often with poorer outcomes.

People with dementia and their carers usually begin help-seeking from close family and then follow this up by consulting primary care physicians.” [7] People from BME populations as a whole have been reported to present later for a number of reasons. These include normalisation of memory problems, concerns about stigma related to dementia, belief that families rather than services are the appropriate resource, previous negative experiences of health services, concern about the threat of receiving a diagnosis, language barriers and lack of knowledge[7–11]. One small study compared eighteen carers of patients with dementia from Black Caribbean or African, South Asian and White backgrounds and found that barriers to help-seeking seemed to be culturally specific [8]. In order to reduce healthcare inequalities, it is important that diverse needs of differing BME groups are identified [12–14]. This would help to contribute to evidence based interventions for specific BME groups. While there have been some initiatives to address these barriers to help-seeking, their outcomes have not been evaluated [6, 15]. No initiative would be universally successful as individuals vary within as well as between groups [13, 16].

While help-seeking for memory problems among minorities as a group has been extensively researched, no-one has attempted to analyse and compare the views of diverse groups of people within Black African and Caribbean British communities. We therefore aimed in this study to explore the barriers to help seeking for memory problems from a large sample of Black African and Caribbean community members and compare common and diverging opinions amongst the varied demographic groups to identify the main themes and use this information to devise, refine and evaluate an intervention to increase timely dementia diagnosis for BACE.

## Method

### Ethical approval

The study was approved by the National Research Ethics Service Committee East of England—Hatfield. All participants gave written informed consent.

### Sample

We purposively recruited Black African and Caribbean participants from community groups and subsequent snowball sampling to achieve a maximum variation sample in terms of socio-demographic characteristics. Participants were healthy adults aged over 18, without a known diagnosis of dementia. Participants were not required to have any knowledge or experience of dementia or dementia services.

### Procedure

Two researchers met community group leaders and members to discuss the study and provide information sheets to potential participants. Additional copies were supplied to be circulated outside the community organisations.

Researchers, SB and MR jointly ran eight focus groups, ranging from 3–8 participants, from community organisations. They also conducted three individual interviews. People were given the choice whether to participate in focus groups or individual interviews. Individual interviews were offered to ensure that people who may not feel comfortable talking in a group setting, were still given the opportunity to participate and contribute. Interviews started early July 2014 and lasted three months. Each participant received a gift voucher as a thank you for their time. Interviews were held in community centres or organisation premises, UCL offices, or for some individual interviews in participant’s home. All sessions lasted approximately 60 minutes and were audio recorded. Recruitment continued until theoretical saturation was reached; that is, no new themes were emerging.

### Interview

Participants completed demographic forms before the interview, asking about their age group, sex, how long they had lived in the UK, current or last occupation and their self defined ethnicity. Following a brief introduction, the interviewers read out a case vignette about Mrs Abrahams, a 70-year old African or Caribbean lady with memory problems (see appendix). The vignette was developed for an earlier study and content validity ascertained by checking with several memory clinic psychiatrists that her symptoms suggested significant memory problems, justifying further investigation for the possibility of dementia [17] (see appendix). We then asked how participants would help if they, a relative or close friend experienced similar problems and what would encourage them to seek help and from where. Researchers facilitated discussions using a topic guide covering questions about attitudes, feelings, beliefs, personal experiences and opinions about memory problems in general.

### Analysis

Interview recordings were transcribed verbatim and we sent participants anonymised transcripts, and invited them to comment on their accuracy and add any further remarks as a method of quality control and validation. Nvivo Software (version 9) was used to manage and analyse the data. SB and MR independently analysed the transcripts using a thematic approach to identify emerging ideas, themes and concepts and produced a thematic coding framework. The researchers then coded the interviews using an iterative process and added new themes generated from initial codes [18]. Discrepancies were resolved by discussion and consensus reached. The two researchers regularly reviewed emerging themes as well as consulting with the rest of the team. We have anonymised all quotations, providing non-specific demographic information.

## Results

### Participants and demographics

Fifty people (30 women and 20 men) participated in the study. They defined their ethnicity as Black African (28), Black Caribbean (14), Black British (7) and Indo-Caribbean (1) with ages ranging from 18 to over 85 years old. They were from a wide variety of different occupational backgrounds including student, cleaner, caterer, telephonist, hairdresser, nurse, sales, project manager and engineer. Ten were born in the UK, and 39 had been living in the UK between 4 and 64 years (mean = 33 years). Ten people (8 women and 2 men) had experience of dementia, four as family carers, four who had been or were being assessed for memory problems and two as support workers. Forty-six (92%) participants were registered with a GP practice. Their sociodemographic characteristics are shown in Table 1.

**Table 1:** Socio-demographic characteristic of participants.

	*Number*	*%*
***Ethnicity***		
Black African	28	56
Black Caribbean	14	28
Black British	7	14
Indo-Caribbean	1	2
***Sex***		
Female	30	60
***Country of birth***		
Jamaica	12	24
UK	10	20
Uganda	6	12
Kenya	4	8
Nigeria	4	8
Zimbabwe	4	8
Guyana	2	4
Congo	1	2
Ghana	1	2
Sierra Leone	1	2
Somalia	1	2
South Africa	1	2
St Vincent	1	2
Sudan	1	2
Unknown	1	2
***Marital status***		
Single	22	44
Married or living with partner	17	34
Separated	4	8
Widowed	3	6
Divorced	1	2
Unknown	3	6
***Religion***		
Christian	34	68
Muslim	7	14
Hindu	1	2
Rastafarian	1	2
No religion	2	4
Other religion	3	6
Unknown	2	4
***Education***		
Secondary	16	32
Post-secondary	32	64
Unknown	2	4
***Currently working***		
Employed	16	32
Unemployed	18	36
Retired	13	26
Student	1	2
Homemaker	1	2
Carer	1	2

### Themes

Five main themes identified obstacles to people seeking health service help for dementia. Some were posed as questions by our interviewees to which they were unsure of or differed about the answers. These were: is forgetfulness indicative of dementia; is dementia an illness of BME communities; should people seek health service help for memory problems; fear of lifestyle changes and confidentiality, privacy and family duty within individual’s culture. We discuss these below in more detail. We have presented themes in Table 2, along with how frequently they were discussed. We searched for variations within these themes according to differing demographic characteristics and detailed in the text when areas of dissimilarities emerged. We found that higher number of Africans reported a lack of knowledge of dementia and greater reliance on community, family and friends for support, advice and care. The majority of participants reporting delaying help-seeking because of fear of what might happen including care home admission were women. Africans in particular repeatedly mentioned not having to consider dementia in their home country or remembering having a structure in place to handle it.

**Table 2:** Barriers to help-seeking for memory problems.

	*N*
**Is forgetfulness important or indicative of dementia?**	
Forgetfulness is very common and a normal part of ageing	23
Forgetfulness can be signs of stress, other difficulties or environmental effect	17
Denying that forgetfulness is a problem	7
**Is dementia an illness of BME communities?**	
Dementia not recognised & masked by strong community support	25*
Lack of knowledge of dementia	26*
**Should people seek health service help for memory problems?**	
People should seek professional help for memory problems	26
But, presentation with memory problems will depend on persistence, severity and impact on everyday life	21
*Wasting GPs’ time and resources*	
Feeling that one should wait until problem becomes severe enough to seek help	18
Accustomed to delay help-seeking because of service cost in home county	7
*Health professionals being unhelpful*, *rushed or dismissive*	
Obstructive GP receptionist	6
Consultation time too short and rushed	18
Poor relationship with GP	16
GP unhelpful and difficult to trust	13
Favour alternative help pathway—private or hospital instead of GP	12
Belief that GP will not be able to help	14
**Fear of lifestyle changes**	
*Negative consequences of help-seeking for memory problems*	
Feeling that nothing can be done	6
Being medicated because GPs are too prompt to prescribe medication	10
Alternative and natural treatments not supported by healthcare	9
General fear of what might happen	14**
Fear of institutionalisation and loss of autonomy	13
**Confidentiality, privacy and family duty within individual’s culture**	
*Avoid stigma*, *pride and embarrassment*	
Perceived stigma of mental illness	23
Embarrassment associated with receiving a diagnosis of mental health	18
*Individual privacy and confidentiality and keeping it within the family*	
Difficulty trusting others with confidential information	5
Personal and legal consequences of information disclosure	3
Accustomed to keep matters private	8
Feeling that care, support and advice lies within the family and close friend circle	37***
Shrinkage of extended family can lead to fear, withdrawal and isolation	21
*Religion and spiritual beliefs*	
Reliance on prayers and spiritual beliefs for solution	11

*17 were Africans.

**12 out of the 14 were female.

***23 were Africans.

#### Is forgetfulness important or indicative of dementia?

Most participants attributed forgetfulness to normal ageing:

*“Where I come from there’s no definition of dementia*., *an old person is starting to forget*, *[it’s] part of growing old*.*”*

(P28, Zimbabwean man, aged 45–54; in UK 14 years)

*“…everybody has that memory loss*, *everyone forgets*, *so they actually think that that phase is actually normal and it also depends on the age*. *So such people will never look for help*.*”*

(P01, Zimbabwean female, aged 45–54; in UK 24 years)

Whilst others attributed memory problems to stress or other life difficulties:

*“…there are a lot of factors*… *it’s a stress of life that can cause her to forget the basic keys and that is*, *maybe she has other stress she’s going through…*.*”*

(P05, Nigerian man, aged 35–44; in UK 9 years)

Nearly all participants knew the term “dementia” but not if or when forgetfulness should be considered indicative of an illness:

*“She was always saying*, *‘Oh I keep forgetting things*, *or you know*, *I can’t get the words out of my mouth’*. *We never recognised it as an issue …”*

(P48, British woman, aged 45–54; UK born)

As a result, recognising the right time to seek help was often problematic:

*“We all accept that we forget things*, *but at which point*, *do you consider that someone needs to seek help*, *that information needs to be available*, *because it’s not there*.*”*

(P44, Zimbabwean woman, aged 55–64; in UK 43 years)

*“I think that over the years there are times when I’ve forgotten things*, *things that I needed to do (*…*) but am I at that stage that*…*that I should get help*?*”*

(P08, Zimbabwean woman, aged 45–54; in UK 15 years)

#### Is dementia an illness of BME communities?

A number of participants explained that their communities would not consider dementia a concern as people where they were born did not see it as an illness. Africans in particular repeatedly mentioned not having to consider dementia in their home country or remembering having a structure in place to handle it, while others thought it only affected their White counterparts:.

*“If I go back to where I was born*, *you see*, *dementia*, *we don’t do dementia in our communities*.”

(P28, Zimbabwean man, aged 45–54; in UK 14 years)

*“When you talk about dementia… this is a White*, *old White peoples disease*, *it’s not seen as Black people have dementia*.*”*

(P49, Jamaican man, aged 45–54; in UK 43 years)

#### Should people seek health service help for memory problems?

Nearly all participants thought Mrs Abrahams should seek help for her memory problems:

*“Do people say no to that*? *Really*? *Oh wow*!*”*

(P16, Black-British man, aged 25–34; UK born)

However, when asked directly whether they would seek help if experiencing similar issues, participants often responded with less certainty as they felt seeking professional help would be dependent upon the persistence, severity and impact upon everyday life of forgetfulness.

*“It depends on how severe the forgetting is or how frequent*.*”*

(P05, Nigerian man, aged 35–44; in UK 9 years)

*“When you get nearly housebound*, *because every time you go out*, *you lose your way*, *then I think*, *that’s when people will consider it to be serious enough*, *to go to the doctor…”*

(P44, Zimbabwean woman, aged 55–64; in UK 43 years)

**Delaying help-seeking until crisis point and participants concern about wasting the GP’s time and resources:** Participants tended to minimise the importance of forgetfulness and thought it was not a serious enough problem to warrant help-seeking.:

*“You just don’t want to go there when you think it could just be a mild problem and you would just feel that you’re wasting the GP’s time*. *You’d be just going there to say*, *oh I forgot this…*?*”*

(P17, British man, aged 25–34; UK born)

*“People go to the GP when they think it’s a serious reason to go to the GP*, *you know Black people*, *we don’t go to the GP until you feel that pain for about a month or so…we will go if it’s really necessary*.*”*

(P16, British man, aged 25–34; UK born)

Some participants were hesitant to seek help because of having previously to pay for healthcare in their countries of origin:

*“In Africa*, *for example*, *the healthcare system is not free*. *So*, *for us it’s like*, *I’ll wait*, *maybe when I’m properly sick and dying*, *then that’s when I’ll go*… *even here*.*”*

(P25, African woman, aged 45–54; in UK 14 years)

The reluctance to seek help until a problem becomes relatively severe was also explained as being due to poverty in the UK or the need to satisfy basic living needs being more pressing than forgetfulness:

*“There are so many problems … to do with poverty*, *poor housing*, *and lack of proper services*. *And something as little as forgetting is not going to be a priority*… *[but] whether they are going to sleep hungry*, *whether they have heating*.*”*

(P24, Kenyan man, aged 35–44; in UK 14 years)

**Health professionals being unhelpful rushed or dismissive:** Participants reported gaining access to GPs was made difficult by practice receptionists; which deterred them from speaking out especially about sensitive matters:

*“Because you’ve got to get past the receptionist first; she’s like a Rottweiler*… *You’ve got to tell her your information and we’re not used to doing that before we get to the doctor*.*”*

(P32, Jamaican woman, aged 55–64; in UK 50 years)

*“A receptionist at the desk starts shouting out to you…What is the reason you’re here*? *The GP’s very busy now… you’re late …If you are suffering with*… *dementia*, *or think you may be … [it] can make you just want to walk out…”*

(P34, Jamaican woman, aged 35–44; UK born)

Participants described the time allocated for GP consultations as insufficient to discuss memory problems. They did not mention being referred to a memory service which had more time:

*“But sometimes you go to the doctor; they don’t even have time for you*. *As you go in they write something*, *you haven’t got time*.*”*

(P41, Jamaican woman, aged 75–84; in UK 55 years)

*“GPs*, *sometimes it is quite hard to sort of open up*… *There’s this quick*, *sort of like factory so like mentality where it’s like ok come in*,. *Ok this is the symptom*, *explain it to me etc*.?*”*

(P18, African man aged 35–44; UK born)

This led to participants’ choices to seek help initially from other pathways

*“Instead of me wasting my time at my doctor’s place*, *because he’s looking at his time*… *I’d rather go to a friend or go for counselling because*, *they would have more time*.*”*

(P23, Nigerian woman, aged 25–34; in UK 10 years)

Many participants were also concerned that GPs would be dismissive. This compounded people’s reluctance to seek help:

”…*there is a tendency for the healthcare professionals to play the issue down and say*, *you know*, *you’ll be all right*, *unless it becomes very serious*.”

(P05, Nigerian man, aged 35–44; in UK 9 years)

#### Fear of Lifestyle changes

There were also thought to be negative consequences of help-seeking for memory problems. There was a reluctance to seek help for dementia due to a lack of perceived benefit and possibility of harm. GPs were also regarded as too prompt to medicate:

*“The damage is already done*. *So*, *I don’t think doctor can help*. *They might prescribe medications*. *That’s not gonna help*. *It might make it get a bit worse*.*”*

(P19, African/Jamaican man, aged 55–64; in UK 48 years)

*“GPs …they don’t really investigate what’s wrong with you; they’ll just write a prescription and it’s always about drugs and… take that and*, *you know*, *go away*, *bye-bye*, *see you later*.*”*

(P30, Jamaican woman, aged 35–44; UK born)

Some people preferred pursuing alternative routes and natural forms of medicine, more akin to systems of medicine in their countries of origin:

*“… because if we’re talking about the older generation*… *coming from the Caribbean or Africa*… *where perhaps a herb*… *heals your cold*…*with the Western Culture … it’s about you know hey have this pill…it’s not really solving the problem*, *it’s just shutting down my acknowledgment of it*.”

(P18, African man, aged 35–44; UK born)

*“So if I had memory issues*… *I’m more likely to exhaust avenues within my own community before I go… we’ve got our own medication that can heal us*.*”*

(P49, Jamaican man, aged 45–54; in UK 43 years)

The fear of loss of autonomy was a barrier to help-seeking for memory problems. Participants feared that receiving a diagnosis, could lead to what they saw as institutionalisation in care homes:

*“The next thing you’re seeing is that big door*, *when you’re institutionalised*, *in a mental home*, *which has got so much bad press*.*”*

(P46, Guyanese woman, aged 65–74; in UK 54 years)

*“…They think that if I ask for help*… *I’m taken away from my own home*. *So really*, *that alone would make someone not to think to ask for help… Therefore it’s better*… *you know [to] do my own things*, *according to my own culture*, *with my own people…*.*”*

(P02, Congolese woman, aged 55–64 in UK 18 years)

In addition, participants were generally concerned about receiving a diagnosis of dementia due to stigma, particularly within their communities:

*“It’s a stigma …*. *In the Black community*, *in particular*, *nobody wants to say that you’re mad*, *in inverted commas*, *not that you’re a little bit senile*.*”*

(P46, Guyanese woman, aged 65–74; in UK 54 years)

*“Sometimes they are scared*, *some they are proud*, *afraid people will laugh at*, *becomes like a stigma for some people*.*”*

(P06, Ugandan woman, aged 65–74; in UK 15 years)

#### Confidentiality, privacy and family duty within individual’s culture

While concerns about confidentiality were related to the health system, they were also part of cultural attitudes to privacy and worry about people’s own status. Some participants expressed a lack of trust in the healthcare systems ability to handle their confidential information appropriately:

*“With all these things going on with the NHS changing and stuff like*, *it’s like how can you trust them*. *You know*, *can you trust them with your confidential information*.*”*

(P23, Nigerian woman, aged 25–34; in UK 10 years)

*“Yes*, *there’s no confidentiality*.*“*

(P29, Kenyan woman, aged 45–54; in UK 28 years)

Concerns about confidentiality were partly related to how disclosure of their personal information could be used against them by official agencies:

*“You don’t want too much people to know your business*. *Because you don’t know how they’re [going to] use it against you*, *as a black person*.*”*

(P23, Nigerian woman, aged 25–34; in UK 10 years)

*“Most African people…fear…to seek help until they reach…a critical point…They are worried to lose their freedom…*.*People come*, *social service*, *sometimes the police*, *immigration*, *all those fear to lose their freedom*.*”*

(P01, Ugandan woman, aged 45–54; in UK 24 years)

On the other hand, some explained these concerns as because privacy is deeply rooted in their cultures and value systems from a young age:

*“We are very secretive*. *It’s very true*.*”*

(P36, Ghanaian man, aged 18–24; UK born)

*“But the secretive*… *is deep-rooted in our culture…From when I was a child it’s*… *don’t tell people your business…Because they’re older people they’re not going to explain why*… *it …become a habit*.*”*

(P35, Jamaican woman, aged 55–64; in UK 47 years)

A strong sense of duty to care for elders within the family and community meant participants delayed help-seeking until they could no longer manage. The family was considered “instead of” not “in addition” to professional help, and linked with a sense of deep cultural respect for elders:

*“If it is a problem*, *as a family member*, *I can get the help*. *… So really*, *I would be thinking that the help is within the family*, *so I wouldn’t look for external help*.*”*

(P08, Zimbabwean woman, aged 45–54; in UK 15 years)

*“… I think sometimes*, *it’s really difficult*, *to assist*… *because they are your parents*, *after all… elders*, *in your family*, *and you respect them*, *and it’s very difficult*, *to actually convince them*, *sometimes*.*”*

(P44, Zimbabwean woman, aged 55–64; in UK 43 years)

Despite strong community networks, some older people reported limited extended family in the UK, as a reason for delayed help-seeking, as this made them feel more vulnerable or less able to seek help:

*“… As you get older -What’s going to happen to you*?… *You just don’t know*. *You haven’t got that family*, *that will support you*… *and you’re really scared*. *The last thing you’re going to do is go to the doctor*, *and say*, *I think I’m going a bit funny in the head*…*”*

(P44, Zimbabwean woman, aged 55–64; in UK 43 years)

*“… So*, *really*, *the early stages for them*, *is very difficult to report it*, *unless it’s an immediate family who’s going to report it*. *And they don’t necessarily live with immediate family*.*”*

(P43, Jamaican man, aged 65–74; in UK 55 years)

#### Religion and spiritual beliefs

A small number of participants suggested that some delayed help-seeking for memory problems because their religion encourages people to initially turn to their faith for help:

*“The church system is now basically discouraging people to go for medical help because everything is spiritual now*. *So if you suffer from an illness pray for it and you’ll be healed*. *that happens a lot in black churches*.*”*

(P36, Ghanaian man, aged 18–24 years; UK born)

*“Yes*, *they don’t go to the doctor*, *they go to the spiritualist*.*”*

(P42, Sierra Leonean woman, aged 65–74; in UK 44 years)

Others felt that people’s strong belief systems could also replace the need for immediate medical help-seeking:

*“…*. *So*, *literally*, *they know there’s something wrong*… *the belief that the person will get better can also override the fact that I need to go and see the doctor*.*”*

(P29, Kenyan woman, aged 45–54; in UK 28 years)

## Discussion

This is the largest qualitative study to investigate barriers to help-seeking for memory problems specifically within the Black African and Caribbean communities. We recruited a maximum variation sample of people from a range of age groups, backgrounds, country of origin, time in the UK, religion and socio-economic background. Some of the barriers to presentation with dementia have been reported before, but others are more specific barriers not cited in the wider BME help-seeking literature.

What we found which was new was that while many people recognised forgetfulness, some thought dementia was a white person’s illness and by implication possibly not a real illness for them. Forgetfulness did not indicate a potential illness that they might have and therefore, there was little point in seeking medical help from a GP for such an unimportant and minor problem. Many thought that help-seeking from a GP was only for severe, pervasive and persistent problems, causing significant interference with daily life and this was true of illness in general, as a result of paying for consultations in their countries of origin. These were new themes which were not recorded in the recent systematic review of barriers to help seeking in dementia and may differ according to the specific BME group, the health system and to the recency of immigration [7]. People also believed the GP has too little time to consider anything as complex as memory and did not appreciate that they could be referred to memory services that would be able to consider their problems in more detail.

While earlier studies of help seeking in dementia had identified negative experiences of the health system [7, 19], we could only find one previous study which had seen the lack of trust which was a common theme across all participants [7]. Some said that people from the Caribbean and Africa are prone to secrecy and highly value privacy of personal affairs and that this is something they are told from childhood. While secrecy has been reported as a consequence of stigma, including self stigma in black African and Caribbean HIV and cancer patients, [20, 21] we do not think it has been reported as a general cultural behaviour not specific to illness or within dementia. Fear, shame and stigma associated with mental illness and dementia within BME communities have been linked to problems being hidden and connected to later presentation [6, 22, 23] This is similar to misperceptions of the difficulty of access to services, and the risk of treatment and disease progression that have been found to be connected to later presentation with HIV of both Black Caribbean but more so amongst Black African migrants [24]. A general feeling of suspicion about patient confidentiality, whether GPs would listen or there is any effective help and whether confidential information could be used against you as a black person are similar to the reasons migrants with HIV give for presenting later to services [25].

Similar to other studies, and as with anxiety and depression, African and Caribbean individuals were less likely to seek help from Western doctors, partly as they believed that doctors simply give tablets and are therefore more likely to seek help for these conditions from religion, a herbalist or self-treatment with herbal remedies [26, 27]. African American people have also been found more likely to see loss of cognitive ability in dementia as a supernatural intervention, an act or punishment from God or a curse [28, 29] However, while religion can hinder access to traditional care pathways, it also assists in a sense of internal positive coping and influences the experience of dementia care received and given [30] it is not however the case that African and Caribbean people with all illness avoid doctors and a recent study in multiple sclerosis found that white and black participants accessed similar amounts of health care [31].

As in earlier research, many participants reported delaying help-seeking for memory problems because of previous negative experiences and perceptions of the health care system, normalisation of symptoms [6, 19], experiences of shame and stigma within the community [5, 19] as well as memory problems being dealt with within the family context first, until symptoms led to a crisis [19]. The stigma for individuals associated with the family knowing about going for help outside the community is similar to African refugee experiences in other Western countries [32].

While we thought that barriers for the older or first generation migrants living in the UK who were not born, nor raised within the culture may no longer be present in UK born or raised participants, we found no evidence of a difference between those born in the UK; those who have lived here for a long time and relatively new immigrants.

### Strengths and weaknesses of research

A large and demographically diverse sample was recruited, ensuring a wide range of perspectives about barriers to help-seeking for memory problems in the Black African and Caribbean communities. The qualitative methodology allowed a detailed picture of barriers to help-seeking for memory problems to be explored in depth.

The sample was drawn predominantly from established community centres and groups, using snowball sampling techniques. Recruitment through community organisations and leaders has been found to be the most successful way of recruiting ethnic minority people to qualitative health research but there is also a risk of community leaders choosing the participants and thus the opinions [33]. We recruited from many different community organisations and thus any effect of choice by the leaders would have been minimised by this heterogeneity. Participants were recruited from inner city, suburban and semi-rural areas which strengthened the generalisability of our findings. However, we did not recruit many people who did not mix within their communities. We are not claiming to generalise our findings to black communities beyond those Black African and Caribbean Communities in Britain. It is vital that BME groups are not treated as homogenous [12] and their diverse needs are identified and considered [34]. Our data does, however, indicate new themes which are worth exploring in other populations, including African Americans.

We used forgetfulness and memory problems interchangeably, but some participants conceptualised the two terms differently; thinking that the word “forgetful” always described a normal phenomenon and therefore could not be a memory problem, said they would respond to them differently in terms of help-seeking behaviour.

### Implications for the future: development of an intervention

More recently, barriers to help-seeking within the South Asian community have been explored and while there are similarities, such as an assumption that forgetfulness is a part of normal ageing or concerns about stigma [17], there are important differences, particularly with regard to the emphasis on privacy and worries about wasting GP time. This indicates that barriers to help-seeking for dementia can be specific for different BME groups and therefore worthy of targeting in an intervention.

Our findings can inform an intervention to overcome some of these barriers, modify attitudes and promote timely help seeking for memory problems in Black African and Caribbean communities. It would stress that dementia is a physical illness, that people from black backgrounds also may develop dementia, and that early presentation with it can lead to better outcomes and is consistent with dutiful family care. In addition, while a GP can not necessarily spend a long time with each individual; they want people with memory problems to come to see them, to discuss with them whether the problem may be symptomatic of something more serious or to treat another cause of the problem. They can appropriately refer people with possible dementia to memory clinics where there will be time available and whose role is to prolong independence and support families in caring.

The intervention should also include key information about dementia to allow people to differentiate it from occasional memory lapses and recognise when to seek professional help. It should allay worries about loss of autonomy, breach of confidentiality and freedom of choice in health decision making. The source of the information should be a recognisable authority and inspire confidence. Despite some misgivings about healthcare services, our interviewees specified the NHS (National Health Service) as a source they were comfortable with, suggesting that distrust may be at individual rather than organisational level. They suggested a logo or NHS delivery channel was considered an effective way to encourage people to treat an intervention as valuable and containing important information. Assurance that healthcare staff would be aware of the intervention and sensitive to people cultural differences were considered an important part of a successful intervention and a stepping-stone to building better relationship between service professionals and members of these communities

Any intervention would require testing to ensure it met its goal of increasing equity of service provision.

## Appendix

### Interview vignette

We want you to think about Mrs Abrahams who is a 70 year old woman. Family members have noted that she is more forgetful lately. She cannot remember conversations with people and forgets appointments with her doctor. She often misplaces important items like her keys and glasses. She is physically healthy but is concerned about her memory.

She’s just a person we’ve made up, but you know, there are people like that, who have similar memory problems. And as we said, she’s forgetting things, she forgets where her glasses and her keys are, and people close to her have noticed the memory problems. And she’s physically healthy, but she’s concerned about her memory. So just to ask all of you, if you knew someone like this, would you think that she should get help for her memory problems.
